# Bis{4-methylbenzyl 2-[4-(propan-2-yl)benzyl­idene]hydrazine­carbodi­thio­ato-κ^2^ *N* ^2^,*S*}nickel(II): crystal structure and Hirshfeld surface analysis

**DOI:** 10.1107/S2056989017002419

**Published:** 2017-02-17

**Authors:** Enis Nadia Md Yusof, Thahira B. S. A. Ravoof, Mohamed I. M. Tahir, Mukesh M. Jotani, Edward R. T. Tiekink

**Affiliations:** a Department of Chemistry, Faculty of Science, Universiti Putra Malaysia, 43400, UPM Serdang, Selangor Darul Ehsan, Malaysia; b Department of Physics, Bhavan’s Sheth R. A. College of Science, Ahmedabad, Gujarat 380001, India; c Research Centre for Crystalline Materials, School of Science and Technology, Sunway University, 47500 Bandar Sunway, Selangor Darul Ehsan, Malaysia

**Keywords:** crystal structure, nickel(II), hydrazine carbodi­thio­ate, Hirshfeld surface analysis

## Abstract

The complete mol­ecule of the title hydrazine carbodi­thio­ate complex, [Ni(C_19_H_21_N_2_S_2_)_2_], is generated by the application of a centre of inversion. The Ni^II^ atom is *N*,*S*-chelated by two hydrazinecarbodi­thio­ate ligands, which provide a *trans*-N_2_S_2_ donor set that defines a distorted square-planar geometry. The conformation of the five-membered chelate ring is an envelope with the Ni^II^ atom being the flap atom. In the crystal, *p*-tolyl-C—H⋯π(benzene-^*i*^Pr), ^*i*^Pr-C—H⋯π(*p*-tol­yl) and π–π inter­actions [between *p*-tolyl rings with inter-centroid distance = 3.8051 (12) Å] help to consolidate the three-dimensional architecture. The analysis of the Hirshfeld surface confirms the importance of H-atom contacts in establishing the packing.

## Abstract 2

Two *N*,*S*-chelating hydrazinecarbodi­thio­ate ligands provide a *trans*-N_2_S_2_ donor set and a distorted square-planar geometry for the Ni^II^ atom. In the crystal, a three-dimensional network is sustained by C—H⋯π and π–π inter­actions.

## Chemical context   

Schiff bases derived from *S*-*R*-di­thio­carbazate (*R* = meth­yl/benz­yl/methyl­benz­yl) and heterocyclic aldehydes or ketones have received much attention in recent years owing to their cytotoxicity (Ali *et al.*, 2002 ▸; Beshir *et al.*, 2008 ▸; Yusof *et al.*, 2015*a* ▸,*b* ▸), as well as their specific and selective anti-bacterial and anti-fungal properties (Low *et al.*, 2014 ▸; Maia *et al.*, 2010 ▸; Pavan *et al.*, 2010 ▸).
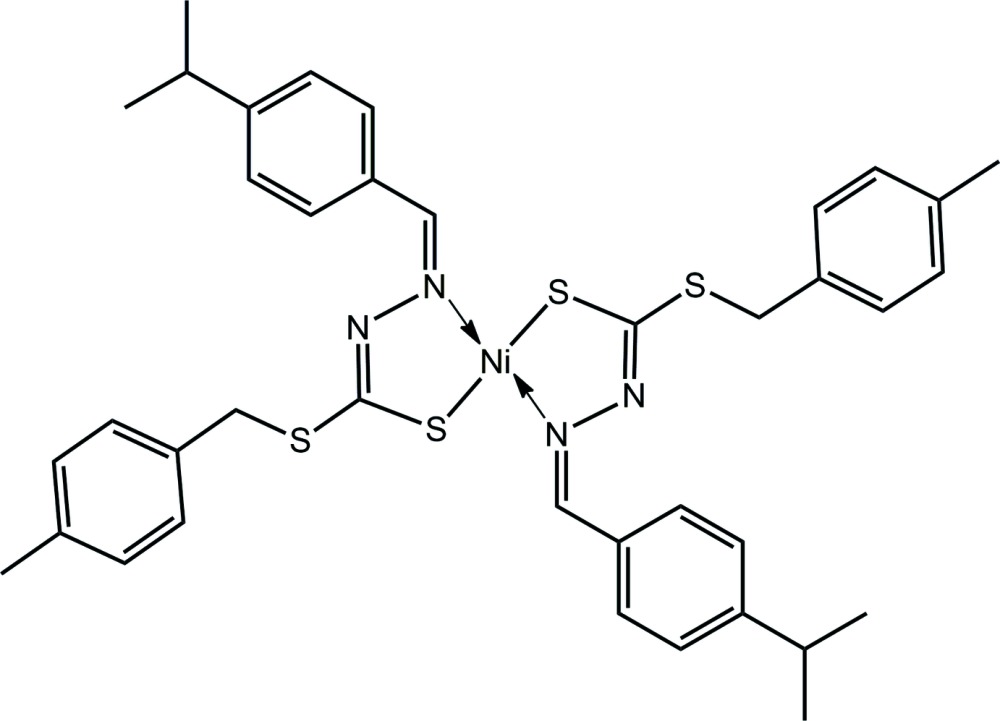


Schiff bases that react with different metal ions often show different types of coordination modes. Metal complexes are versatile mol­ecules with a wide range of pharmacological properties due to the inherent characteristics of both the central metal atoms and ligands (Meggers, 2009 ▸). Various transition metal complexes have been reported to induce DNA cleavage by attacking the sugar or base moieties of DNA through the formation of reactive oxygen species (ROS) (Burrows & Muller, 1998 ▸). A nickel(II) bis-di­thio­carbazate complex has been used in the photo-catalytic production of hydrogen as a catalyst (Wise *et al.*, 2015 ▸). Nickel(II) di­thio­carbazate has also been reported to have non-linear optical (NLO) properties (Liu *et al.*, 2016 ▸) with the potential to be used in signal processing (Bort *et al.*, 2013 ▸; Hales *et al.*, 2014 ▸), ultrafast optical communication, data storage, optical limiting (Price *et al.*, 2015 ▸; Bouit *et al.*, 2007 ▸), optical switching (Gieseking *et al.* 2014 ▸; Thorley *et al.*, 2008 ▸), logic devices and bio-imaging (Ahn *et al.*, 2012 ▸; Zhu *et al.*, 2016 ▸). In line with our inter­est in evaluating the structures of different isomeric di­thio­carbazate Schiff bases and their metal complexes, we report herein the synthesis of the title complex, (I), its X-ray crystal structure determination and a detailed study of the supramolecular association by an analysis of its Hirshfeld surface.

## Structural commentary   

**Figure 1:**
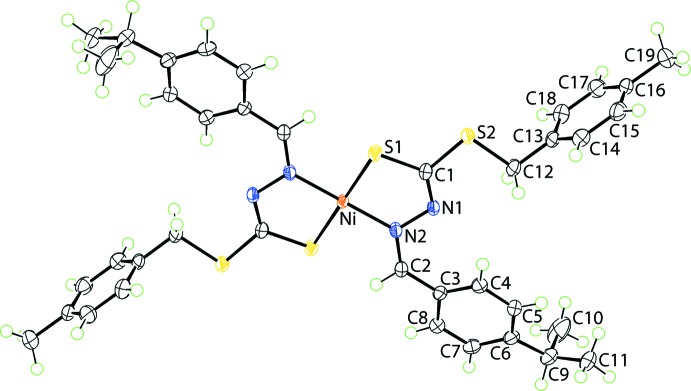
The mol­ecular structure of (I), showing the atom-labelling scheme and displacement ellipsoids at the 70% probability level. The Ni^II^ atom is situated on a centre of inversion. Unlabelled atoms are related by the symmetry operation (1 − *x*, 1 − *y*, 1 − *z*).

The Ni^II^ atom in (I), Fig. 1 ▸, is located on a crystallographic centre of inversion and is coordinated by two *S*,*N*-chelating hydrazinecarbodi­thio­ate anions. From symmetry, the resulting N_2_S_2_ donor set has like atoms *trans*, and the square-planar coordination geometry is strictly planar. Distortions from the ideal geometry are related to the deviations of angles subtended at nickel by the donor atoms, Table 1 ▸. The C1—N1—N2—C2 backbone of the ligand exhibits a twist as seen in the value of the torsion angle, *i.e*. −165.61 (17)°. Despite being involved in a formal bond to the Ni^II^ atom, the C1—S1 bond length of 1.7296 (19) Å is still significantly shorter than those formed by the S2 atom, *i.e*. C1—S2 = 1.7479 (18) Å and C12—S2 = 1.824 (2) Å.

The planarity of the N_2_S_2_ donor set does not extend to the five-membered chelate ring, which has an envelope conformation with the nickel atom lying 0.465 (2) Å above the least-squares plane through the remaining atoms [r.m.s. deviation = 0.0016 Å]. The sequence of C1=N1, N1—N2 and N2=C2 bond lengths of 1.294 (2), 1.408 (2) and 1.300 (2) Å, respectively, suggests limited conjugation across this residue. Each of the benzene rings of the S- and N-bound substituents is twisted with respect to the least-squares plane through the chelate ring. Thus, a nearly orthogonal relationship exists between the chelate and *p*-tolyl rings, with the dihedral angle being 89.72 (5)°. Less dramatic is the twist of the ^*i*^Pr-substituted ring with the dihedral angle being 13.83 (9)°. The dihedral angle between the aromatic rings is 84.31 (6)°.

## Supra­molecular features   

**Figure 2:**
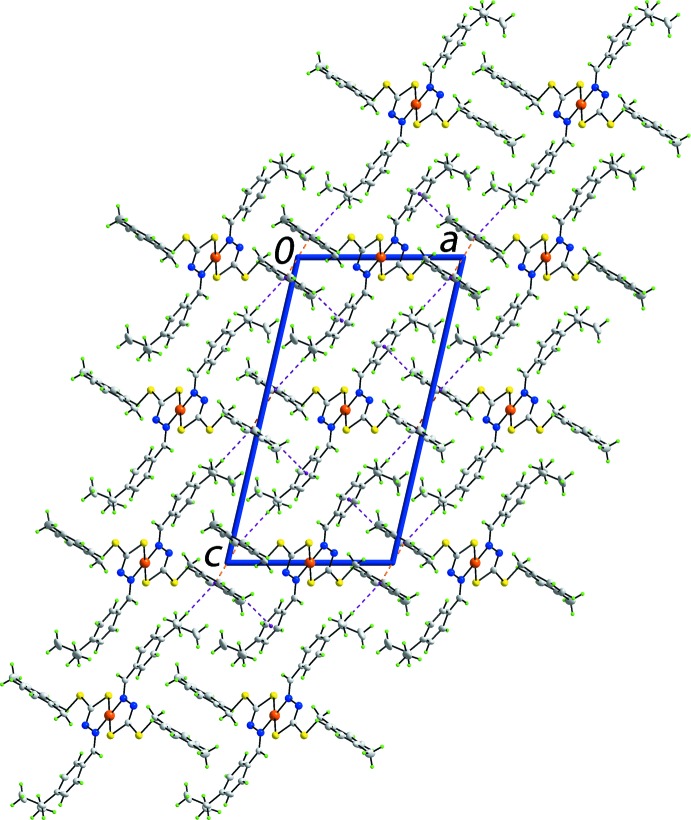
The mol­ecular packing in (I): a view of the unit-cell contents shown in projection down the *b* axis. The π–π and C—H⋯π inter­actions are shown as orange and purple dashed lines, respectively.

The two sites potentially available for hydrogen bonding in (I), *i.e*. the S1 and N1 atoms, are involved in intra­molecular inter­actions, Table 2 ▸. The only discernible contacts in the crystal involve π-systems (Spek, 2009 ▸). Thus, each of the independent rings is involved in C—H⋯π contacts, *i.e. p*-tolyl-C—H⋯π(^*i*^Pr-benzene) and ^*i*^Pr-benzene-C—H⋯π(*p*-tol­yl) contacts, Table 2 ▸. In addition, centrosymmetrically related *p*-tolyl rings self-associate *via* face-to-face, π–π, inter­actions [inter-centroid distance = 3.8051 (12) Å for symmetry operation −*x*, −1 − *y*, 1 − *z*], indicating the *p*-tolyl ring participates in two distinct inter­actions. The result of the supra­molecular association is the formation of a three-dimensional architecture, Fig. 2 ▸.

## Analysis of the Hirshfeld surfaces   

**Figure 3:**
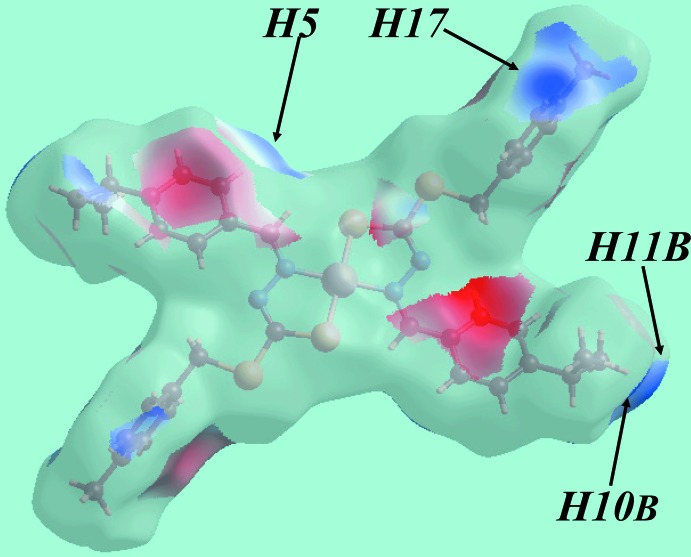
A view of the Hirshfeld surface for (I) mapped over the electrostatic potential over the range ±0.025 au.

**Figure 4:**
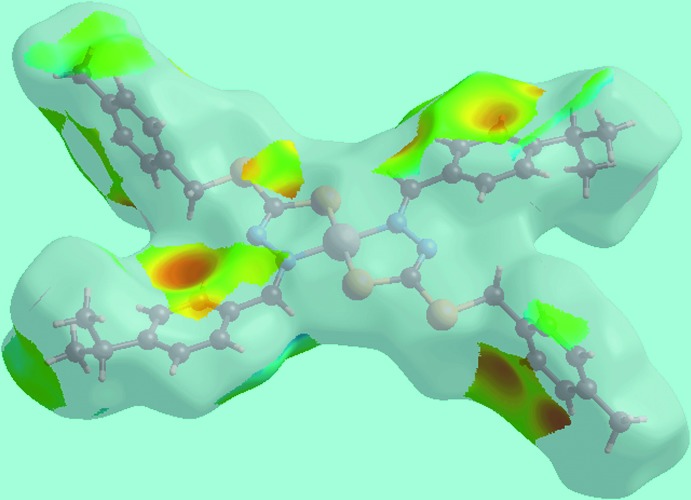
The view of the Hirshfeld surface mapped over *d*_e_. The bright-orange spots near rings indicate their involvement in C—H⋯π inter­actions.

**Figure 5:**
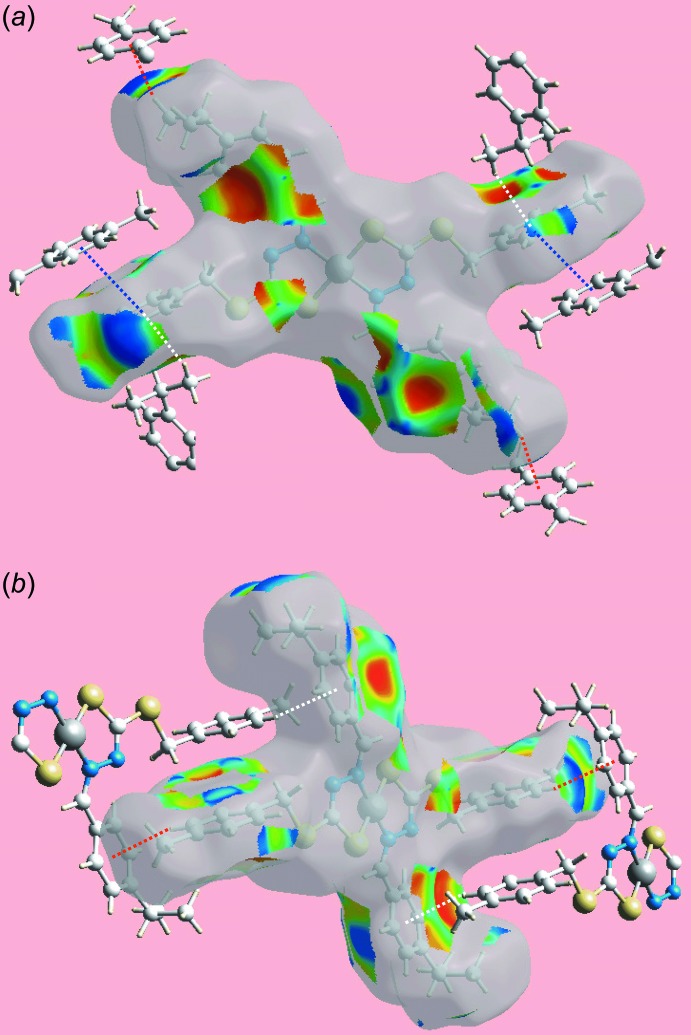
Two views (*a*) and (*b*) of the Hirshfeld surface mapped with shape-index property about a reference mol­ecule. The C—H⋯π and π⋯H—C inter­actions in both views are indicated with red and white dotted lines, respectively. The blue dotted lines in (*a*) indicate π–π stacking between *p*-tolyl rings.

The Hirshfeld surface analysis for (I) was performed as described in a recent publication of a heavy-atom structure (Mohamad *et al.*, 2017 ▸). The non-appearance of characteristic red spots on the Hirshfeld surface mapped over *d*_norm_ (not shown) clearly indicates the absence of conventional hydrogen bonding in the crystal. The donors and acceptors of C—H⋯π inter­actions, involving atoms of each of the ^*i*^Pr-benzene and *p*-tolyl rings, are viewed as blue and light-red regions and correspond to the respective positive and negative potentials on the Hirshfeld surface mapped over electrostatic potential (over the range ± 0.025 au), Fig. 3 ▸. The acceptors of the C—H⋯π inter­actions are also viewed as bright-orange spots appearing near ^*i*^Pr-benzene and *p*-tolyl rings on the Hirshfeld surface mapped over *d*_e_, Fig. 4 ▸. The immediate environment about a reference mol­ecule within the Hirshfeld surface mapped with shape-index property is illustrated in Fig. 5 ▸. The C—H⋯π and their reciprocal contacts, *i.e*. π⋯H—C contacts, between ^i^Pr–H11*B* and the *p*-tolyl ring are represented by red and white dotted lines, respectively in Fig. 5 ▸ *a*; the blue dotted lines in Fig. 5 ▸ *a* represent π–π stacking between *p*-tolyl rings at −*x*, −1 − *y*, 1 − *z*. The other C—H⋯π contacts involving *p*-tolyl-H17 and ^*i*^Pr-benzene rings are illustrated in Fig. 5 ▸ *b*.

**Figure 6:**
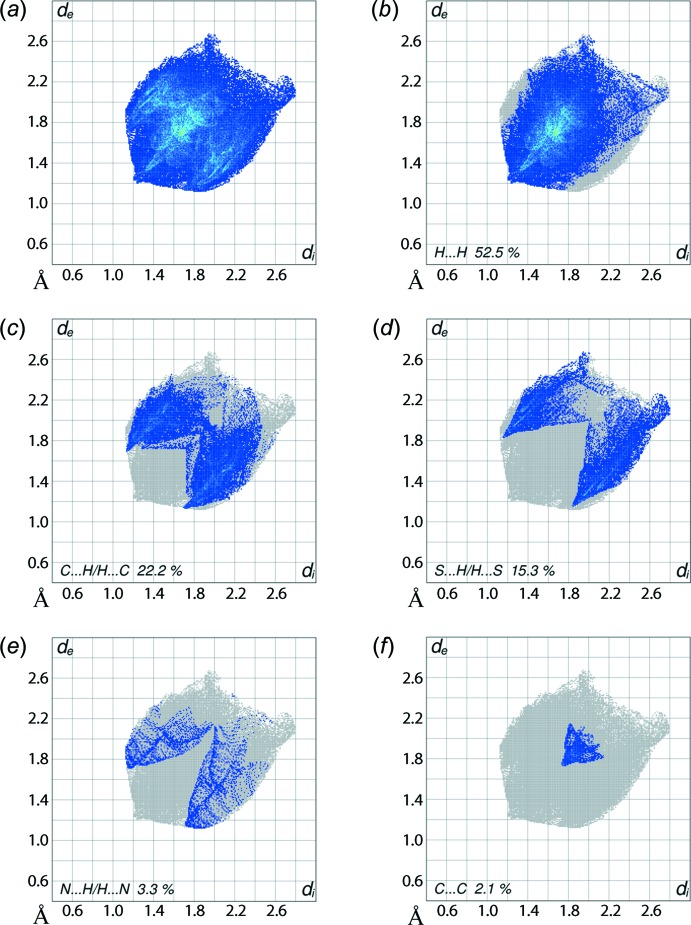
The two-dimensional fingerprint plots for (I): (*a*) all inter­actions, and delineated into (*b*) H⋯H, (*c*) C⋯H/H⋯C, (*d*) S⋯H/H⋯S, (*e*) N⋯H/H⋯N and (*f*) C⋯C inter­actions.

The overall two-dimensional fingerprint plot and those delineated into H⋯H, C⋯H/H⋯C, S⋯H/H⋯S and N⋯H/H⋯N and C⋯C contacts (McKinnon *et al.*, 2007 ▸) illustrated in Fig. 6 ▸ *a*–*f*. From the qu­anti­tative summary of the relative contributions of the various inter­atomic contacts given in Table 3 ▸, it is important to note the dominant contribution of hydrogen atoms to the Hirshfeld surface, *i.e*. 95.3%. In the fingerprint plot delineated into H⋯H contacts. Fig. 6 ▸ *b*, the points are distributed in the major part of the plot, but they do not make significant contributions to the mol­ecular packing as their inter­atomic separations are greater than sum of their van der Waals radii, *i.e. d*_e_ + *d*_i_ > 2.4 Å. The presence of short inter­atomic C⋯H/H⋯C contacts, see Table 4 ▸, and C—H⋯π inter­actions contribute to the second largest contribution to the Hirshfeld surface, *i.e*. 22.2%. This is consistent with the fingerprint plot, Fig. 6 ▸ *c*, where the short inter­atomic C⋯H/H⋯C contacts appear as a pair of small peaks at *d*_e_ + *d*_i_ ∼ 2.8 Å and also as the blue regions around the participating hydrogen atoms, namely H5 and H10*B*, on the Hirshfeld surface mapped over electrostatic potential, Fig. 3 ▸. The involvement of the chelating S1 and N1 atoms in intra­molecular inter­actions, Table 2 ▸, prevents them from forming inter­molecular S⋯H/H⋯S and N⋯H/H⋯N contacts. However, the symmetrical distribution of points with the usual characteristics in their respective plots, Fig. 6 ▸ *d* and *e*, indicate meaningful contributions to the Hirshfeld surface, Table 3 ▸. A small, *i.e*. 2.1%, but recognizable contribution from C⋯C contacts to the Hirshfeld surface is ascribed to π–π stacking inter­actions between symmetry-related *p*-tolyl rings, and appear as an arrow-like distribution of points around *d*_e_ = *d*_i_ 1.9 Å in Fig. 6 ▸ *f*. The other contacts have low percentage contributions to the surface and are likely to have negligible effects on the mol­ecular packing, Table 3 ▸.

## Database survey   

**Figure 7:**
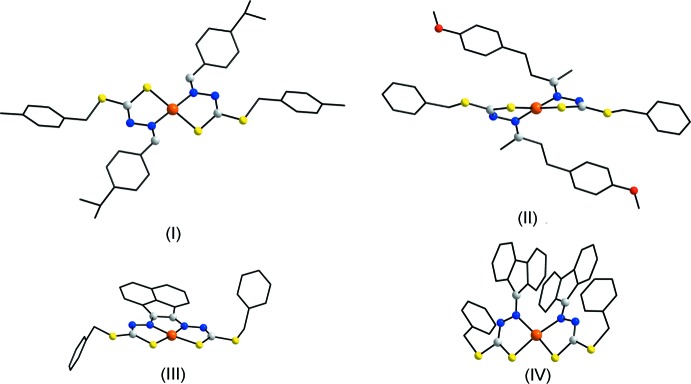
Simplified mol­ecular structure diagrams of (I)–(IV). All C atoms, except those of the C—N—N—C backbone, are represented as small black spheres and H atoms have been omitted.

There are three closely related nickel(II) di­thio­carbazate complexes in the crystallographic literature (Groom *et al.*, 2016 ▸); these are illustrated in simplified form in Fig. 7 ▸. Complex (II) differs from (I) only in the nature of the terminal substituents (Tan *et al.*, 2012 ▸). Despite there being only small differences in chemical composition, a distinct coordination geometry is observed, with the Ni^II^ atom located on a twofold rotation axis and the N_2_S_2_ donor set having *cis*-dispositions of like atoms. In (III), with a formal link between the two imine functionalities, the *cis*-N_2_S_2_ arrangement is imposed by the geometric requirements of the bis­(di­thio­carbazate) di-anion (Zhou *et al.*, 2002 ▸). The mol­ecular structure of (IV), again with a *cis*-N_2_S_2_ donor set, appears to indicate that steric effects do not preclude a *cis*-N_2_S_2_ coordination geometry (Liu *et al.*, 2000 ▸). With the foregoing in mind, it appears that the mol­ecular structure of (I) is unprecedented, suggesting further systematic investigations in this area are warranted.

## Synthesis and crystallization   

The *S*-4-methyl­benzyl­dithio­carbazate (S4MDTC) precursor was synthesized by following a procedure adapted from the literature (Omar *et al.*, 2014 ▸). The Schiff base was also synthesized using a procedure adapted from the literature (Yusof *et al.*, 2015*b* ▸) by the reaction of S4MDTC (2.12 g, 0.01 mol), dissolved in hot aceto­nitrile (100 ml), with an equimolar amount of 4-iso­propyl­benzaldehyde (1.48 g, 0.01 mol) in absolute ethanol (20 ml). The mixture was then heated at 353 K until half of the mixture solution reduced and allowed to cool to room temperature until a precipitate formed. The compound was recrystallized from ethanol solution and dried over silica gel.

The synthesized Schiff base (0.33 g, 1 mmol) was dissolved in hot aceto­nitrile (50 ml) and added to nickel(II) acetate tetra­hydrate (0.13 g, 0.5 mmol) in an ethano­lic solution (30 ml). The mixture was heated and stirred to reduce the volume of the solution. Precipitation occurred once the mixture cooled to room temperature. The precipitate then was filtered and dried over silica gel. The complex was recrystallized from its methanol solution. Brown prismatic crystals were formed from the filtrate after being left to stand for a month. The crystals were filtered and washed with absolute ethanol at room temperature. Yield: 70%. M.p.: 479–480 K. Elemental composition calculated for C_38_H_42_N_4_NiS_4_: C, 61.53; H, 5.71; N, 7.55. Found: C, 61.67; H, 5.87; N, 7.55%. FT–IR (ATR, cm^−1^): 1589, ν(C=N); 997, ν(N—N); 823, ν(C=S).

## Refinement   

Crystal data, data collection and structure refinement details are summarized in Table 5 ▸. The carbon-bound H-atoms were placed in calculated positions (C—H = 0.95–0.99 Å) and were included in the refinement in the riding-model approximation, with *U*_iso_(H) set to 1.2–1.5*U*_eq_(C).

## Supplementary Material

Crystal structure: contains datablock(s) I, global. DOI: 10.1107/S2056989017002419/hb7658sup1.cif

Structure factors: contains datablock(s) I. DOI: 10.1107/S2056989017002419/hb7658Isup2.hkl

CCDC reference: 1532446

Additional supporting information:  crystallographic information; 3D view; checkCIF report

## Figures and Tables

**Table 1:** Selected bond lengths (Å)

Ni—S1	2.1747 (5)	C1—S2	1.7479 (18)
Ni—N2	1.9137 (15)	C12—S2	1.824 (2)
C1—S1	1.7296 (19)		

**Table 2:** Hydrogen-bond geometry (Å, °) *Cg*1 and *Cg*2 are the centroids of the (C3–C8) and (C13–C18) rings, respectively.

*D*—H⋯*A*	*D*—H	H⋯*A*	*D*⋯*A*	*D*—H⋯*A*
C2—H2⋯S1^i^	0.95	2.48	3.0691 (17)	120
C4—H4⋯N1	0.95	2.40	2.865 (2)	110
C17—H17⋯*Cg*1^ii^	0.95	2.84	3.761 (2)	164
C11—H11*B*⋯*Cg*2^iii^	0.98	2.96	3.880 (3)	158

Symmetry codes: (i) 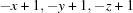; (ii) 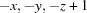; (iii) 
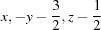
.

**Table 3:** Percentage contribution of the different inter­molecular contacts to the Hirshfeld surface in (I)

Contact	% contribution
H⋯H	52.5
C⋯H/H⋯C	22.2
S⋯H/H⋯S	15.3
N⋯H/H⋯N	3.3
C⋯C	2.1
Ni⋯H/H⋯Ni	2.0
S⋯N/N⋯S	1.8
C⋯S/S⋯C	0.4
S⋯S	0.3
C⋯N/N⋯C	0.1

**Table 4:** Short inter­atomic contacts in (I)

Contact	distance	symmetry operation
C16⋯H10*B*	2.84	*x*, − 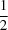 − *y*, − 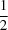 + *z*
C19⋯H5	2.88	-*x*, −1 − *y*, 1 − *z*

**Table 5:** Experimental details

Crystal data	
Chemical formula	[Ni(C_19_H_21_N_2_S_2_)_2_]
*M*_r_	741.70
Crystal system, space group	Monoclinic, *P*2_1_/*c*
Temperature (K)	100
*a*, *b*, *c* (Å)	11.5799 (7), 7.3910 (3), 21.9848 (16)
β (°)	103.033 (7)
*V* (Å^3^)	1833.1 (2)
*Z*	2
Radiation type	Mo *K*α
μ (mm^−1^)	0.79
Crystal size (mm)	0.30 × 0.20 × 0.10

Data collection	
Diffractometer	Agilent Xcalibur Eos Gemini
Absorption correction	Multi-scan (*CrysAlis PRO*; Agilent, 2011 ▸)
*T*_min_, *T*_max_	0.895, 1.000
No. of measured, independent and observed [*I* > 2σ(*I*)] reflections	8467, 4192, 3393
*R*_int_	0.030
(sin θ/λ)_max_ (Å^−1^)	0.674

Refinement	
*R*[*F* ^2^ > 2σ(*F* ^2^)], *wR*(*F* ^2^), *S*	0.035, 0.085, 1.02
No. of reflections	4192
No. of parameters	217
H-atom treatment	H-atom parameters constrained
Δρ_max_, Δρ_min_ (e Å^−3^)	0.48, −0.24

Computer programs: *CrysAlis PRO* (Agilent, 2011 ▸), *SHELXS97* (Sheldrick, 2008 ▸), *SHELXL2014* (Sheldrick, 2015 ▸), *ORTEP-3 for Windows* (Farrugia, 2012 ▸), *DIAMOND* (Brandenburg, 2006 ▸) and *publCIF* (Westrip, 2010 ▸).

## supplementary crystallographic information

### Crystal data

**Table:**

[Ni(C_19_H_21_N_2_S_2_)_2_]	*F*(000) = 780
*M**_r_* = 741.70	*D*_x_ = 1.344 Mg m^−^^3^
Monoclinic, *P*2_1_/*c*	Mo *K*α radiation, λ = 0.7107 Å
*a* = 11.5799 (7) Å	Cell parameters from 3077 reflections
*b* = 7.3910 (3) Å	θ = 2.3–28.7°
*c* = 21.9848 (16) Å	µ = 0.79 mm^−^^1^
β = 103.033 (7)°	*T* = 100 K
*V* = 1833.1 (2) Å^3^	Prism, brown
*Z* = 2	0.30 × 0.20 × 0.10 mm

### Data collection

**Table:**

Agilent Xcalibur Eos Gemini diffractometer	4192 independent reflections
Radiation source: Enhance (Mo) X-ray Source	3393 reflections with *I* > 2σ(*I*)
Graphite monochromator	*R*_int_ = 0.030
Detector resolution: 16.1952 pixels mm^-1^	θ_max_ = 28.6°, θ_min_ = 2.3°
ω scans	*h* = −15→14
Absorption correction: multi-scan (CrysAlis PRO; Agilent, 2011)	*k* = −9→9
*T*_min_ = 0.895, *T*_max_ = 1.000	*l* = −27→29
8467 measured reflections	

### Refinement

**Table:**

Refinement on *F*^2^	Primary atom site location: structure-invariant direct methods
Least-squares matrix: full	Hydrogen site location: inferred from neighbouring sites
*R*[*F*^2^ > 2σ(*F*^2^)] = 0.035	H-atom parameters constrained
*wR*(*F*^2^) = 0.085	*w* = 1/[σ^2^(*F*_o_^2^) + (0.0367*P*)^2^ + 0.7493*P*] where *P* = (*F*_o_^2^ + 2*F*_c_^2^)/3
*S* = 1.02	(Δ/σ)_max_ = 0.001
4192 reflections	Δρ_max_ = 0.48 e Å^−^^3^
217 parameters	Δρ_min_ = −0.24 e Å^−^^3^
0 restraints	

### Special details

**Table:**

Geometry. All esds (except the esd in the dihedral angle between two l.s. planes) are estimated using the full covariance matrix. The cell esds are taken into account individually in the estimation of esds in distances, angles and torsion angles; correlations between esds in cell parameters are only used when they are defined by crystal symmetry. An approximate (isotropic) treatment of cell esds is used for estimating esds involving l.s. planes.

### Fractional atomic coordinates and isotropic or equivalent isotropic displacement parameters (Å^2^)

**Table:**

	*x*	*y*	*z*	*U*_iso_*/*U*_eq_	
Ni	0.5000	0.5000	0.5000	0.01242 (9)	
S1	0.46625 (4)	0.27399 (6)	0.43507 (2)	0.01691 (12)	
S2	0.29415 (4)	−0.01653 (6)	0.43496 (2)	0.01892 (12)	
N1	0.36782 (13)	0.1991 (2)	0.53197 (7)	0.0153 (3)	
N2	0.43911 (13)	0.3485 (2)	0.55606 (7)	0.0141 (3)	
C1	0.37730 (16)	0.1603 (2)	0.47593 (9)	0.0150 (4)	
C2	0.45497 (16)	0.3715 (2)	0.61599 (9)	0.0156 (4)	
H2	0.5057	0.4692	0.6325	0.019*	
C3	0.40706 (16)	0.2706 (2)	0.66191 (9)	0.0158 (4)	
C4	0.37058 (16)	0.0885 (3)	0.65720 (9)	0.0173 (4)	
H4	0.3835	0.0158	0.6237	0.021*	
C5	0.31580 (17)	0.0154 (3)	0.70149 (9)	0.0191 (4)	
H5	0.2931	−0.1084	0.6981	0.023*	
C6	0.29286 (17)	0.1173 (3)	0.75084 (9)	0.0192 (4)	
C7	0.33687 (18)	0.2946 (3)	0.75775 (9)	0.0215 (4)	
H7	0.3273	0.3649	0.7925	0.026*	
C8	0.39416 (17)	0.3687 (3)	0.71472 (9)	0.0197 (4)	
H8	0.4252	0.4879	0.7210	0.024*	
C9	0.21599 (19)	0.0493 (3)	0.79346 (10)	0.0247 (5)	
H9	0.2445	0.1068	0.8353	0.030*	
C10	0.0885 (2)	0.1130 (4)	0.76693 (13)	0.0426 (7)	
H10A	0.0864	0.2455	0.7655	0.064*	
H10B	0.0371	0.0696	0.7937	0.064*	
H10C	0.0604	0.0646	0.7247	0.064*	
C11	0.2185 (2)	−0.1560 (3)	0.80247 (11)	0.0298 (5)	
H11A	0.1814	−0.2148	0.7629	0.045*	
H11B	0.1749	−0.1879	0.8343	0.045*	
H11C	0.3009	−0.1968	0.8159	0.045*	
C12	0.21972 (18)	−0.1039 (3)	0.49369 (9)	0.0202 (4)	
H12A	0.2782	−0.1605	0.5283	0.024*	
H12B	0.1800	−0.0041	0.5110	0.024*	
C13	0.12954 (16)	−0.2422 (3)	0.46281 (9)	0.0167 (4)	
C14	0.15875 (17)	−0.4252 (3)	0.46395 (9)	0.0195 (4)	
H14	0.2363	−0.4633	0.4841	0.023*	
C15	0.07536 (19)	−0.5521 (3)	0.43589 (10)	0.0227 (4)	
H15	0.0966	−0.6764	0.4372	0.027*	
C16	−0.03838 (18)	−0.5005 (3)	0.40599 (10)	0.0212 (4)	
C17	−0.06739 (17)	−0.3171 (3)	0.40435 (10)	0.0230 (4)	
H17	−0.1447	−0.2791	0.3838	0.028*	
C18	0.01567 (17)	−0.1900 (3)	0.43244 (10)	0.0211 (4)	
H18	−0.0054	−0.0656	0.4309	0.025*	
C19	−0.1304 (2)	−0.6381 (3)	0.37581 (11)	0.0329 (5)	
H19A	−0.1946	−0.6411	0.3982	0.049*	
H19B	−0.0936	−0.7580	0.3775	0.049*	
H19C	−0.1626	−0.6045	0.3322	0.049*	

### Atomic displacement parameters (Å^2^)

**Table:**

	*U*^11^	*U*^22^	*U*^33^	*U*^12^	*U*^13^	*U*^23^
Ni	0.01367 (16)	0.01287 (16)	0.01220 (17)	−0.00309 (12)	0.00605 (13)	−0.00017 (13)
S1	0.0214 (2)	0.0164 (2)	0.0156 (2)	−0.00518 (18)	0.00956 (19)	−0.00249 (19)
S2	0.0239 (2)	0.0188 (2)	0.0157 (2)	−0.00893 (18)	0.0080 (2)	−0.0039 (2)
N1	0.0169 (8)	0.0147 (7)	0.0157 (8)	−0.0057 (6)	0.0062 (6)	−0.0015 (7)
N2	0.0141 (7)	0.0131 (7)	0.0160 (8)	−0.0033 (6)	0.0054 (6)	−0.0013 (6)
C1	0.0160 (9)	0.0122 (8)	0.0174 (9)	−0.0010 (7)	0.0049 (8)	0.0020 (7)
C2	0.0154 (9)	0.0155 (9)	0.0165 (10)	−0.0035 (7)	0.0049 (8)	0.0003 (8)
C3	0.0146 (9)	0.0205 (9)	0.0127 (9)	−0.0035 (7)	0.0037 (7)	0.0012 (8)
C4	0.0185 (9)	0.0198 (9)	0.0144 (9)	−0.0022 (7)	0.0052 (8)	−0.0012 (8)
C5	0.0202 (9)	0.0200 (9)	0.0169 (10)	−0.0057 (7)	0.0040 (8)	0.0018 (8)
C6	0.0187 (9)	0.0255 (10)	0.0133 (9)	−0.0029 (8)	0.0037 (8)	0.0027 (8)
C7	0.0275 (11)	0.0258 (10)	0.0126 (9)	−0.0035 (8)	0.0074 (8)	−0.0033 (8)
C8	0.0218 (10)	0.0214 (10)	0.0159 (10)	−0.0051 (8)	0.0040 (8)	0.0001 (8)
C9	0.0275 (11)	0.0317 (11)	0.0167 (10)	−0.0059 (9)	0.0089 (9)	0.0021 (9)
C10	0.0313 (13)	0.0582 (17)	0.0461 (16)	0.0062 (11)	0.0250 (12)	0.0199 (14)
C11	0.0330 (12)	0.0345 (12)	0.0240 (11)	−0.0100 (10)	0.0109 (10)	0.0080 (10)
C12	0.0232 (10)	0.0227 (10)	0.0173 (10)	−0.0079 (8)	0.0101 (8)	−0.0014 (8)
C13	0.0170 (9)	0.0199 (9)	0.0156 (9)	−0.0052 (7)	0.0086 (8)	−0.0010 (8)
C14	0.0199 (10)	0.0203 (9)	0.0189 (10)	0.0007 (8)	0.0059 (8)	0.0015 (8)
C15	0.0326 (11)	0.0165 (9)	0.0207 (10)	−0.0021 (8)	0.0094 (9)	0.0000 (8)
C16	0.0260 (10)	0.0230 (10)	0.0159 (10)	−0.0113 (8)	0.0074 (8)	−0.0010 (8)
C17	0.0155 (9)	0.0301 (11)	0.0225 (11)	−0.0019 (8)	0.0025 (8)	0.0032 (9)
C18	0.0222 (10)	0.0174 (9)	0.0251 (11)	−0.0004 (8)	0.0080 (9)	0.0024 (9)
C19	0.0376 (13)	0.0351 (12)	0.0251 (12)	−0.0209 (10)	0.0051 (10)	−0.0034 (10)

### Geometric parameters (Å, º)

**Table:**

Ni—S1	2.1747 (5)	C9—H9	1.0000
Ni—N2	1.9137 (15)	C10—H10A	0.9800
Ni—N2^i^	1.9138 (15)	C10—H10B	0.9800
Ni—S1^i^	2.1746 (5)	C10—H10C	0.9800
C1—S1	1.7296 (19)	C11—H11A	0.9800
C1—S2	1.7479 (18)	C11—H11B	0.9800
C12—S2	1.824 (2)	C11—H11C	0.9800
N1—C1	1.294 (2)	C12—C13	1.509 (2)
N1—N2	1.408 (2)	C12—H12A	0.9900
N2—C2	1.300 (2)	C12—H12B	0.9900
C2—C3	1.461 (3)	C13—C18	1.391 (3)
C2—H2	0.9500	C13—C14	1.393 (3)
C3—C8	1.405 (3)	C14—C15	1.387 (3)
C3—C4	1.408 (3)	C14—H14	0.9500
C4—C5	1.386 (3)	C15—C16	1.386 (3)
C4—H4	0.9500	C15—H15	0.9500
C5—C6	1.395 (3)	C16—C17	1.395 (3)
C5—H5	0.9500	C16—C19	1.514 (3)
C6—C7	1.401 (3)	C17—C18	1.386 (3)
C6—C9	1.516 (3)	C17—H17	0.9500
C7—C8	1.385 (3)	C18—H18	0.9500
C7—H7	0.9500	C19—H19A	0.9800
C8—H8	0.9500	C19—H19B	0.9800
C9—C11	1.529 (3)	C19—H19C	0.9800
C9—C10	1.534 (3)		
			
N2—Ni—N2^i^	180.00 (7)	C9—C10—H10A	109.5
N2—Ni—S1^i^	93.71 (5)	C9—C10—H10B	109.5
N2^i^—Ni—S1^i^	86.29 (5)	H10A—C10—H10B	109.5
N2—Ni—S1	86.30 (5)	C9—C10—H10C	109.5
N2^i^—Ni—S1	93.70 (5)	H10A—C10—H10C	109.5
S1^i^—Ni—S1	180.0	H10B—C10—H10C	109.5
C1—S1—Ni	94.14 (6)	C9—C11—H11A	109.5
C1—S2—C12	101.14 (9)	C9—C11—H11B	109.5
C1—N1—N2	111.31 (15)	H11A—C11—H11B	109.5
C2—N2—N1	114.86 (15)	C9—C11—H11C	109.5
C2—N2—Ni	126.01 (13)	H11A—C11—H11C	109.5
N1—N2—Ni	119.11 (12)	H11B—C11—H11C	109.5
N1—C1—S1	125.12 (14)	C13—C12—S2	108.11 (14)
N1—C1—S2	120.09 (14)	C13—C12—H12A	110.1
S1—C1—S2	114.77 (11)	S2—C12—H12A	110.1
N2—C2—C3	130.15 (17)	C13—C12—H12B	110.1
N2—C2—H2	114.9	S2—C12—H12B	110.1
C3—C2—H2	114.9	H12A—C12—H12B	108.4
C8—C3—C4	117.91 (18)	C18—C13—C14	118.53 (17)
C8—C3—C2	115.80 (16)	C18—C13—C12	120.86 (17)
C4—C3—C2	126.26 (18)	C14—C13—C12	120.60 (17)
C5—C4—C3	119.90 (18)	C15—C14—C13	120.43 (18)
C5—C4—H4	120.1	C15—C14—H14	119.8
C3—C4—H4	120.1	C13—C14—H14	119.8
C4—C5—C6	122.24 (18)	C16—C15—C14	121.13 (19)
C4—C5—H5	118.9	C16—C15—H15	119.4
C6—C5—H5	118.9	C14—C15—H15	119.4
C5—C6—C7	117.45 (18)	C15—C16—C17	118.47 (18)
C5—C6—C9	122.87 (18)	C15—C16—C19	121.53 (19)
C7—C6—C9	119.53 (18)	C17—C16—C19	120.00 (19)
C8—C7—C6	120.95 (19)	C18—C17—C16	120.55 (18)
C8—C7—H7	119.5	C18—C17—H17	119.7
C6—C7—H7	119.5	C16—C17—H17	119.7
C7—C8—C3	121.12 (18)	C17—C18—C13	120.87 (18)
C7—C8—H8	119.4	C17—C18—H18	119.6
C3—C8—H8	119.4	C13—C18—H18	119.6
C6—C9—C11	114.35 (18)	C16—C19—H19A	109.5
C6—C9—C10	108.19 (18)	C16—C19—H19B	109.5
C11—C9—C10	110.02 (19)	H19A—C19—H19B	109.5
C6—C9—H9	108.0	C16—C19—H19C	109.5
C11—C9—H9	108.0	H19A—C19—H19C	109.5
C10—C9—H9	108.0	H19B—C19—H19C	109.5
			
C1—N1—N2—C2	−165.61 (17)	C4—C3—C8—C7	−6.2 (3)
C1—N1—N2—Ni	15.80 (19)	C2—C3—C8—C7	172.25 (17)
N2—N1—C1—S1	0.5 (2)	C5—C6—C9—C11	30.3 (3)
N2—N1—C1—S2	−178.25 (12)	C7—C6—C9—C11	−154.27 (19)
Ni—S1—C1—N1	−12.72 (17)	C5—C6—C9—C10	−92.6 (2)
Ni—S1—C1—S2	166.05 (9)	C7—C6—C9—C10	82.8 (2)
C12—S2—C1—N1	−3.28 (18)	C1—S2—C12—C13	171.91 (13)
C12—S2—C1—S1	177.88 (11)	S2—C12—C13—C18	−86.5 (2)
N1—N2—C2—C3	−2.5 (3)	S2—C12—C13—C14	93.3 (2)
Ni—N2—C2—C3	176.01 (15)	C18—C13—C14—C15	−0.6 (3)
N2—C2—C3—C8	−152.0 (2)	C12—C13—C14—C15	179.55 (19)
N2—C2—C3—C4	26.3 (3)	C13—C14—C15—C16	0.2 (3)
C8—C3—C4—C5	4.7 (3)	C14—C15—C16—C17	0.4 (3)
C2—C3—C4—C5	−173.58 (18)	C14—C15—C16—C19	−179.4 (2)
C3—C4—C5—C6	1.2 (3)	C15—C16—C17—C18	−0.5 (3)
C4—C5—C6—C7	−5.6 (3)	C19—C16—C17—C18	179.2 (2)
C4—C5—C6—C9	169.86 (18)	C16—C17—C18—C13	0.0 (3)
C5—C6—C7—C8	4.1 (3)	C14—C13—C18—C17	0.5 (3)
C9—C6—C7—C8	−171.54 (18)	C12—C13—C18—C17	−179.67 (19)
C6—C7—C8—C3	1.8 (3)		

Symmetry code: (i) −*x*+1, −*y*+1, −*z*+1.

### Hydrogen-bond geometry (Å, º)

*Cg*1 and *Cg*2 are the centroids of the (C3–C8) and (C13–C18) rings, respectively.

**Table:**

*D*—H···*A*	*D*—H	H···*A*	*D*···*A*	*D*—H···*A*
C2—H2···S1^i^	0.95	2.48	3.0691 (17)	120
C4—H4···N1	0.95	2.40	2.865 (2)	110
C17—H17···*Cg*1^ii^	0.95	2.84	3.761 (2)	164
C11—H11*B*···*Cg*2^iii^	0.98	2.96	3.880 (3)	158

Symmetry codes: (i) −*x*+1, −*y*+1, −*z*+1; (ii) −*x*, −*y*, −*z*+1; (iii) *x*, −*y*−3/2, *z*−1/2.
